# Quantitative data from six years (2013-2018) of light trap sampling of macromoths (Lepidoptera) in Mt. Hallasan National Park, South Korea

**DOI:** 10.3897/BDJ.8.e51490

**Published:** 2020-04-07

**Authors:** Sei-Woong Choi, Sang-Hyeon Na

**Affiliations:** 1 Mokpo National University, Muan, South Korea Mokpo National University Muan South Korea

## Abstract

**Background:**

This paper presents the results of long-term monitoring of macromoth communities in Mt. Hallasan National Park, South Korea. This mountain shows an altitudinal gradient of vegetation from evergreen deciduous to boreal trees, harbouring more than 550 species of vascular plants. The goal of this project was to investigate the changes in moth assemblages along the altitudinal gradient in this mountain ecosystem. We monitored macromoth communities at 11 sites in Mt. Hallasan National Park from 2013 to 2018, during which time moths were collected once a month from May to October, using an ultraviolet bucket trap. The generated dataset, which represented 587 species and 13,249 individuals from 14 families, can be adopted to establish a baseline for development of a network-orientated database to assess temporal and spatial changes of moths in temperate and tropical forests.

**New information:**

This is the first long-term sampling-event dataset on macromoth assemblages in changing vegetation from evergreen deciduous to boreal tree zones, conducted in Mt. Hallasan National Park, the national park at the highest elevation and located on the largest volcanic island in South Korea. The aim of this study was to provide a description and a link to published data in the format of a peer-reviewed journal and to provide recognition of the effort in a scholarly article (based on data paper definition published at https://www.gbif.org/en/data-papers).

## Introduction

Island ecosystems are self-maintaining entities with well-defined geographical limits and are the combined products of geography (area, latitude, altitude, isolation), ecology (geology, biotope availability, history, land use and management), biology (mobility, colonisation capability, presence of organisms) and time. Thus, island biota are considered ‘individuals’ carrying unique information regarding complex interactions amongst biological, geographical and historical factors (Vitousek et al. 1995, [Bibr B5527539][Bibr B5527062]). However, a number of difficulties, inherent to examining biological diversity and ecosystem functioning on islands, exist due to the less diverse and disharmonious ecosystems and the varying degree of anthropogenic alteration (Vitousek et al. 1995). Montane species on islands are often endemic to a single mountain range and are vulnerable to climate change because they tend to occur in small populations, isolated from other source populations, climatically restricted and limited from moving to higher elevations upon reaching the summit of the mountain (Meyer et al. 2015).

Lepidoptera are one of the mega-diversity insect groups, comprised of more than 160,000 species that play important roles as herbivores and pollinators in terrestrial ecosystems. They also act as a food source for birds and bats and a vital linkage in the food chain between plants and higher trophic organisms. Due to their diversity, easy sampling with a light trap and known habitat associations, moths are considered one of the most suitable insect groups for assessing species diversity against changes in landscape change and management (Alison et al. 2017, Dirzo et al. 2014, Hallmann et al. 2019, Kamikura and Sakata 2019, Kitching et al. 2000, Macgregor et al. 2016, Summerville et al. 2004).

The aim of this study was to investigate the diversity and changes in macromoth communities at 11 sites in Mt. Hallasan National Park, South Korea, over a period of six years (2013-2018). We sampled macromoths to monitor their long-term changes in an island’s mountain ecosystem. The elevational gradient along Mt. Hallasan National Park has resulted in vertical stratification of vegetation zones from boreal to evergreen deciduous, producing a unique biodiversity pattern (Kang 2006, Kong 2007). Elevation gradients on mountains have the potential to enhance our understanding of the impact of climate change on biological communities. Thus, the diversity and distribution of montane species will be a baseline for development of a network-orientated database to assess species responses to climate change in temperate and tropical forests.

## Project description

### Title

Long-term monitoring of macromoths in the southern mountains of South Korea

### Personnel

Sei-Woong Choi and Sang-Hyeon Na

### Study area description

**Figure 1.**
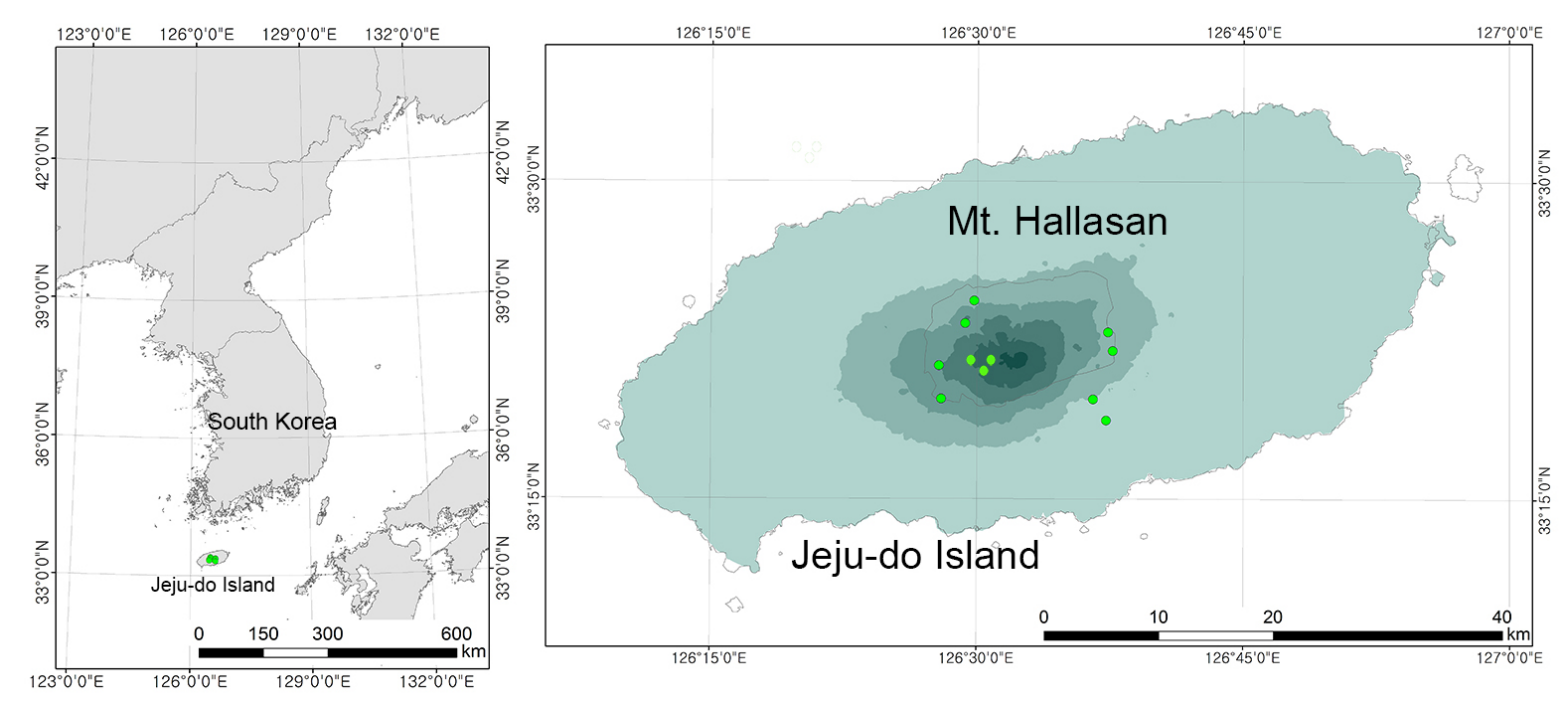
Map of the survey sites in Mt. Hallasan, Jeju-do, South Korea.

Mt. Hallasan National Park (highest peak 1,950 m above sea level, total area 149 km^2^), one of South Korea’s 22 National Parks, is located on the nation’s largest volcanic island, Jeju-do (126°09'42"–126°56'57" E, 33°11'27"–33°33'50" N, 1,825 km^2^, Fig. 1). The annual average temperature of Jeju-do Island is 5.3-10.9°C in areas more than 600 m above sea level and 15.2–16.2°C in coastal areas and the annual precipitation is 2,968-4,746 mm in areas more than 600 m above sea level and 1,095-1,851 mm in coastal areas (Kang 2006).

The vegetation on Mt. Hallasan is comprised of four zones: alpine zone (> 1,800 m a.s.l.), subalpine zone (1,500–1,800 m), temperate deciduous tree zone (400–1,500 m) and evergreen deciduous tree zone (600 m in the southern aspect and 400 m in the northern aspect) (Kong 2007). The alpine zone is characterised by dwarf trees (*Taxus cuspidata* Sieb. & Zucc., *Betula ermani* Chamisso) and shrubs (Diapenis lapponica var. obovata F. Schmidt, *Vaccinium uliginosum* L., Empetrum nigrum var. japonicum L. Koch., Juniperus chinensis var. sargentii Henry, Rhododendron mucronulatum var. ciliatum Nakai). The subalpine zone is characterised by conifers (*Abies koreana* Wilson, *Taxus cuspidata* Sieb. & Zucc.) and deciduous trees (*Betula ermani* Chamisso). The temperate deciduous tree zone is covered with deciduous trees, such as *Quercus serrata* Thunb., *Q. acuta* Thunb., *Q. glauca* Thunb., *Carpinus laxiflora* (Sieb. & Zucc.) Blume, *C. tschonoskii* Maxim., *Acer palmatum* Thunb., *Daphniphyllum macropodum* Miq. and Castanopsis cuspidata var. siebildii Nakai. Evergreen deciduous trees, such as *Cinnamomum campora* Sieb., *Machilus thunbergii* Sieb. & Zucc., *Quercus myrsinaefolia* Bl. and *Camellia japonica* L., are commonly observed at low altitudes. About 550 species of vascular plants are distributed on Mt. Hallasan amongst 1,800 plants found on Jeju-do Island (Kong 2007).

### Funding

National Research Foundation of Korea (2018R1D1A1B07046637)

## Sampling methods

### Study extent

Geographic coverage: Survey areas for collecting moths comprised evergreen deciduous and subalpine tree zones (Table 1

### Sampling description

Sampling method: An ultraviolet light bucket trap, consisting of a 22 Watt ultraviolet circline light tube with a 12 V battery (BioQuip Co., USA), was employed to collect moths at each survey site. Moth sampling was conducted for five hours after dusk. To minimise sampling bias, we sampled moths simultaneously at all 11 sites. Traps were emptied the morning after collection and insects were brought to the lab for identification. Moths were identified at species level using taxonomic literature (Kim et al. 2001, Kononenko et al. 1998, Shin 2001, Kim et al. 2016). Vouchers of collected specimens were deposited in the collection of the Laboratory of Environmental Education, Mokpo National University, South Korea.

## Geographic coverage

### Description

Survey areas for collecting moths comprised evergreen deciduous and subalpine tree zones

### Coordinates

33-18 and 33-24 Latitude; 126-37 and 126-27 Longitude.

## Taxonomic coverage

### Description

Macromoths targeted for this study comprised the moth families that traditionally fall under the category of macrolepidoptera (Kristensen and Skalski 1999), plus two easily identified microlepidoptera families: Bombycidae, Drepanidae, Erebidae, Geometridae, Noctuidae, Nolidae, Notodontidae, Limacodidae, Lasiocampidae, Sphingidae, Saturniidae, Thyrididae, Uraniidae and Zygaenidae.

## Temporal coverage

### Notes

We sampled moths once a month from May to October from 2013 to 2018.

## Usage rights

### Use license

Creative Commons Public Domain Waiver (CC-Zero)

## Data resources

### Data package title

Six years of data (2013-2018) of macromoths (Lepidoptera) in Mt. Hallasan National Park, Republic of Korea

### Resource link

https://datadryad.org/stash/share/wdwhCjEWJ7yNQXzhuwHcN48O45pGOtaTvyvf4SbRnhY

### Number of data sets

3

### Data set 1.

#### Data set name

Data_jejudo_taxaa

#### Data format

csv

#### Number of columns

4

#### Character set

UTF-8

#### 

**Data set 1.** 

Column label	Column description
ID	ID number
Taxon	Species name used in data file (Data-Jejudo-data.csv)
Family	Family name for each species
Species in full name	Genus, species, author and publication year

### Data set 2.

#### Data set name

Data_Jejudo_data.csv

#### Data format

csv

#### Number of columns

5

#### Character set

UTF-8

#### 

**Data set 2.** 

Column label	Column description
Site	Eleven survey site code
Site code	Site abbreviation
Date	Collection date (yyyy-mm-dd)
Taxon	Species
Number of individuals	Number of individuals collected

### Data set 3.

#### Data set name

Data_Jejudo_site_information

#### Data format

csv

#### Number of columns

6

#### Character set

UTF-8

#### 

**Data set 3.** 

Column label	Column description
Site code	Eleven survey site code
Site abbreviation in English	Site abbreviation for site code
Elevation (m)	Elevation above sea level for each survey site
Latitude (N)	Geographic latitude (WG84)
Longitude (E)	Geographic longitude (WG84)
Vegetation type	Dominant vegetation type for each survey site

## Additional information

Suppl. material 1: The total number of moths collected at 11 sites on Mt. Hallasan represented 587 species and 13,249 individuals from 14 families. Amongst the sites surveyed, the total number of species was highest at site JJ_4 (293 species) and the total number of individuals was highest at site JJ_3 (2738 individuals) (Table 2,Table 3).

**Figure 2.**
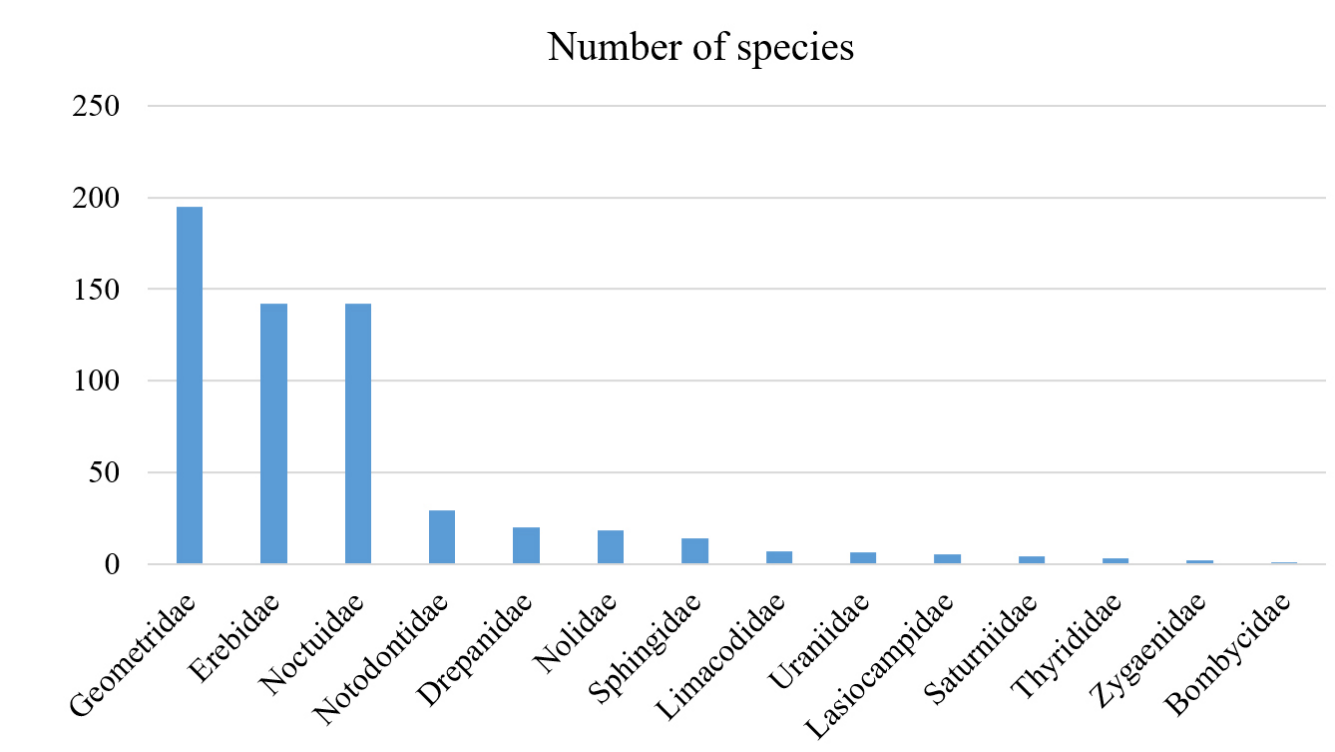
Number of moth species in each family collected from 11 sites in Mt. Hallasan National Park from 2013 to 2018.

**Figure 3.**
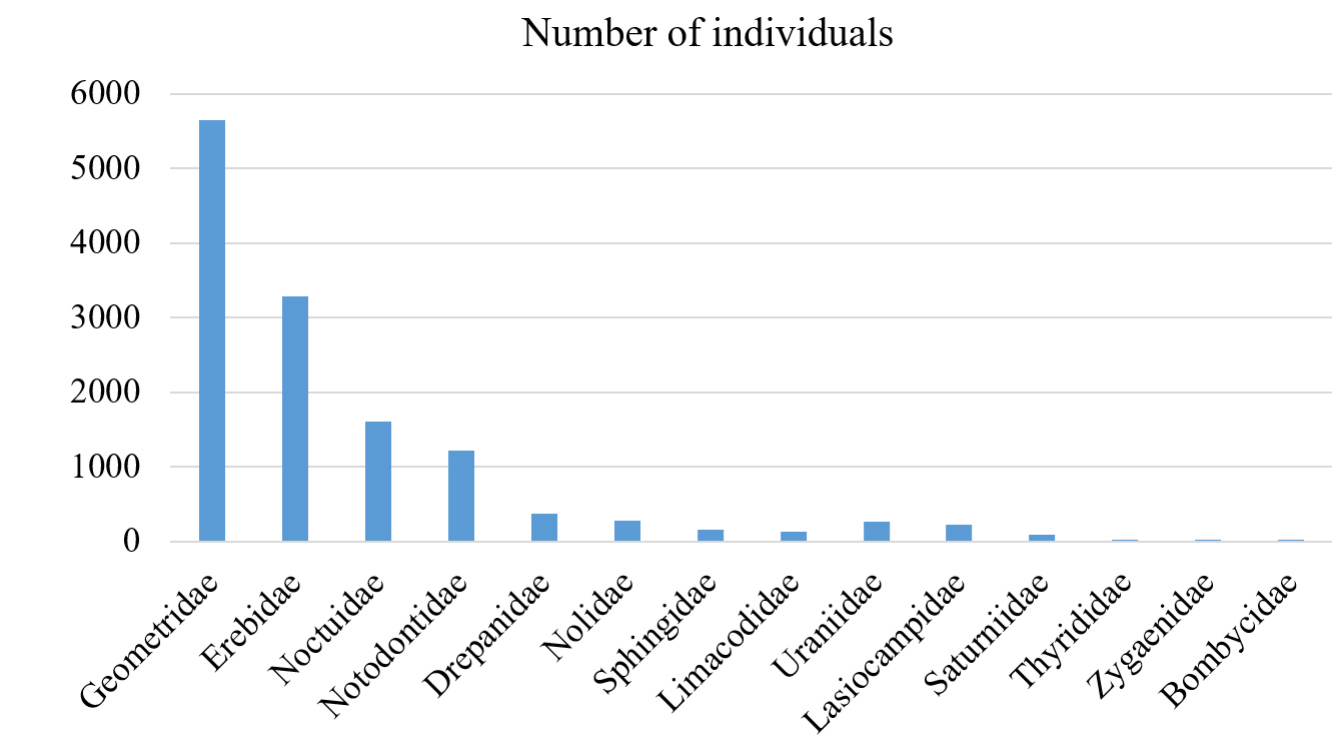
Number of moth individuals in each family collected from 11 sites in Mt. Hallasan National Park from 2013 to 2018.

The family Geometridae was dominant in the total number of species (33%) and in the total number of individuals (42%) (Figs 2, 3). The three families, Geometridae, Erebidae, and Noctuidae, comprised most of the samples: 81.6% of the total species and 79.5% of the total individuals. On the other hand, moths of the Bombycidae and Zygaenidae families represented one and two species, respectively.

A geometrid species, *Alcis angulifera* was dominant with 1,618 individuals, occurring at all survey sites. In addition, five species *Hydrillodes morosa, Ghoria gigantean, Lomographa temerata, Idaea biselata* and *Diarsia pacifia* occurred at all survey sites.

## Supplementary Material

9C04B58F-2D41-51E4-B0CB-D44CFF049B96 10.3897/BDJ.8.e51490.suppl1 Supplementary material 1 Six years of data (2013-2018) of macromoths (Lepidoptera) in Mt. Hallasan National Park, Republic of Korea Data type occurrences File: oo_398032.zip https://binary.pensoft.net/file/398032 Choi, SW, Na, SH

## Figures and Tables

**Table 1.** Site information on Mt. Hallasan National Park, South Korea

**Site Code**	**Local site name**	**Elevation**	**Latitude (N)**	**Longitude (E)**	**Vegetation**
JJ_1	HRR (L)	278 m	33°18′57.0″	126°37′09.9″	Evergreen
JJ_2	HRR (H)	525 m	33°19′56.7″	126°36′25.7″	Evergreen
JJ_3	SPA (H)	752 m	33°22′14.0″	126°37′31.6″	Temperate deciduous
JJ_4	CWS	673 m	33°24′36.1″	126°29′43.3″	Temperate deciduous
JJ_5	SPA (L)	645 m	33°23′06.7″	126°37′16.0″	Temperate deciduous
JJ_6	YS (L)	963 m	33°19′57.6″	126°27′52.6″	Temperate deciduous
JJ_7	ERM	954 m	33°23′31.6″	126°29′13.0″	Temperate deciduous
JJ_8	1100top	1109 m	33°21′32.1″	126°27′44.4″	Temperate deciduous
JJ_9	SJB	1410 m	33°22′32.2″	126°29′58.8″	Subalpine
JJ_10	YS (H)	1630 m	33°21′31.3″	126°30′29.1″	Subalpine
JJ_11	USOR	1699 m	33°21′43.5″	126°31′10.0″	Subalpine

**Table 2.** Numbers of families, species and individuals collected in Mt. Hallasan National Park, South Korea, from 2013 to 2018.

**Site**	**Number of families**	**Number of species**	**Number of individuals**
JJ_1	12	221	814
JJ_2	12	206	922
JJ_3	13	248	2,738
JJ_4	11	293	1,763
JJ_5	13	216	1,600
JJ_6	11	185	2,011
JJ_7	11	225	1,598
JJ_8	11	174	997
JJ_9	10	110	457
JJ_10	7	57	216
JJ_11	7	37	133
Total	14	587	13,249

**Table 3.** Yearly summary of numbers of species and individuals collected from 2013 to 2018 in Mt. Hallasan National Park, South Korea.

**Year**	**Number of families**	**Number of species**	**Number of individuals**
2013	12	243	1,526
2014	11	254	2,255
2015	11	236	2,037
2016	13	248	1,671
2017	12	315	2,996
2018	13	330	2,764
Total	14	587	13,249
